# Allele and transcriptome mining in *Gossypium hirsutum* reveals variation in candidate genes at genetic loci affecting cotton fiber quality and textile flammability

**DOI:** 10.1186/s12870-025-06306-2

**Published:** 2025-03-10

**Authors:** Gregory N. Thyssen, Wayne Smith, Marina Naoumkina, Ganesh Pinnika, Johnie N. Jenkins, Jack C. McCarty, Ping Li, Christopher B. Florane, Don C. Jones, David D. Fang

**Affiliations:** 1 Cotton Fiber Bioscience & Utilization Research Unit, USDA-ARS, Southern Regional Research Center, New Orleans, LA USA; 2 Department of Soil and Crop Sciences, Texas A&M University, College Station, TX USA; 3 Genetics and Sustainable Agriculture Research Unit, USDA-ARS, Mississippi State, MS USA; 4 Cotton Incorporated, Cary, NC USA

**Keywords:** Cotton diversity, Cotton fiber genes, Fiber strength, Transcript mining, Allele mining, Cotton transcription, Nuclear export signal

## Abstract

**Background:**

Breeding valuable traits in crop plants requires identifying diverse alleles in the germplasm that are likely to affect desirable characteristics. The genetic diversity of historic cultivars of cotton is a reservoir of potentially important genes for crop improvement and genetic research. Diversity in the characteristics of harvested cotton fibers affects their suitability for end-use applications. Candidate loci and genes have been identified that affect the length, strength, and maturity of cotton fibers which affect the quality and value of the yarn, thread and textile. Natural genetic mechanisms in the plant may also affect the flammability of the produced textiles.

**Results:**

Here we show that a combination of allele mining and transcriptome analysis can identify candidate genes for cotton fiber traits including strength and perhaps flammability. We found novel DNA variants in fiber-expressed gene families in 132 newly sequenced cotton varieties and identified genes with genotype-specific RNA expression.

**Conclusions:**

Among these, we identified novel variation in DNA sequence and RNA expression in genes at major QTL qD04-ELO-WLIM (JGI-Gohir.D04G160000), qA13-MIC (Gohir.A13G157500), qA07-STR (Gohir.A07G191600), supported the candidacy of qD11-UHML-KRP6 (Gohir.D11G197900) and qD13-STR (Gohir.D13G17450), and identified an additional A03-WLIM transcription factor gene (Gohir.A03G182100) and several RNA expression variant candidates of potential flammability genes that may be useful for plant biologists and cotton breeders. Candidate genes for traits like flame resistance that are likely due to the combination of many small effect QTL can benefit from this multi-mining approach. We provide an annotated variant call format (vcf) file with variations at 24,996 loci that are predicted to affect 10,418 cotton fiber genes in the historic breeding germplasm.

**Supplementary Information:**

The online version contains supplementary material available at 10.1186/s12870-025-06306-2.

## Introduction

Cotton is a source of natural, renewable fibers for textile production and other applications. Since the cotton fiber cell is the most highly elongated plant cell, it is a useful model system for studies of cell polarity and directional cell growth [1–4]. Genetic loci that influence cotton fiber properties, as measured by a high-volume instrument (HVI), have been identified. The length, strength (STR), micronaire (MIC), length uniformity index (UI) and elongation (ELO) are measured for each bale of cotton produced in the United States of America. Micronaire is related to fiber fineness and maturity, and ELO is a measure of how far a fiber will stretch before breaking [5–7]. Candidate genes for contrasting fiber phenotypes have revealed mechanisms of fiber development that are controlled by transcription factors and result in altered cytoskeletal organization in the developing fiber cells [1, 8–11]. Several of the HVI traits are affected by secondary cell wall structure [11, 12]. The WLIM1a family of transcription factors has been shown to both orchestrate cotton fiber transcription and bind to F-actin, a key component of the cytoskeleton [8].

Cotton research extends from ‘dirt to shirt.’[13] Agronomic traits including yield, fiber quality, herbicide tolerance, pest resistance and responses to abiotic environmental stress have been characterized. The genetic factors that likely contribute to these phenotypes have also been identified [14–20]. Beyond these traits that are important to growers, are traits that affect the value of the end-use product.

In addition to the HVI-measured traits that affect the quality and properties of thread, yarn and textiles, end-use consumers are justifiably concerned about textile flammability. Significant progress has been made on the development of effective and safe chemical flame retardants for use on clothing, furniture, upholstery etc. [21, 22]. To complement these chemical additive approaches, we previously identified novel cotton lines that have reduced flammability. Earlier, we had identified a structural variant in the promoter of a key transcription factor gene GhTT2_A07 (Gohir.A07G020200, Gh_A07G2341) in the anthocyanin pathway in brown-colored fiber lines with natural flame resistance [23–26]. More recently, through transgressive segregation, a combination of alleles bred a self-extinguishing white fiber phenotype that is not present in any of the eleven white-fiber parent lines that all lack the GhTT2_A07 brown-fiber allele variant and rapidly burn like all prior tested white-fiber cotton lines [27]. Here, we identified these alleles and novel alleles of related genes in the diversity of the cotton germplasm.

Here we show that a combination of allele and transcriptome mining and prior knowledge can use ‘reverse genetics’ to reveal novel candidate genes in *Gossypium hirsutum* (L.). To accomplish this, we identify diverse allelic variants of fiber expressed genes in the germplasm and breeding lines. The identification of genetic lines based on ‘reverse genetics’ on DNA variation is called ‘Allele Mining.’ [28] Variation in RNA expression in a diverse population at different developmental points is also indicative of a gene that potentially controls a phenotype. This strategy of analysis and selection is termed ‘Transcriptome Mining.’[29].

Further, we present variants in 10,418 fiber expressed genes that we identified in 132 newly sequenced diverse and historic breeding material of *G. hirsutum* lines. We identified variants in genes that are closely related to those that have been described to be involved in cotton fiber development and implicated in textile flammability.

## Materials and Methods

### Plant materials and DNA sequencing

A set of 132 diverse *G. hirsutum* lines and genotypes [Supplemental Table S1] were grown in a greenhouse in New Orleans, LA, USA in 2020. Young leaves from ten seedling plants from each line were collected, immediately frozen and stored at −80 °C. The genomic DNA was extracted [30] and sent to commercial vendors for Illumina HiSeq paired end short read sequencing to 20 × coverage.

### Transcriptome RNA sequencing

Transcriptome sequences of developing fibers were obtained from a previously developed MAGIC population derived from eleven of the lines that are included in this historic germplasm, namely Acala Ultima, Coker 315, Deltapine Acala 90, Fibermax 966, M240-RNR, Paymaster HS 26, Phytogene PSC 355, Stoneville 474, Stoneville 825, Suregrow 747, and Tamcot Pyramid. After 55 half-diallele crosses, five generations of random matings and six generations of single seed descent, a MAGIC population of 550 RILs was established [31–33]. Plants of the 550 MAGIC RILs, about 8 – 12 plants per RIL, were grown in the fields of New Orleans in 2019, 2021, and 2022. Due to field size, not all RILs were planted each year and were each grown once [Supplemental Table S2]. The variability of RNA expression across planting years was examined by ANOVA to confirm the consistency of the environment [Supplemental Tables S3, S4]. Flowers were tagged on the day of anthesis (DOA), and about a dozen developing bolls were collected at 8- and 16- days post anthesis (DPA). Developing bolls were put on ice and transferred to the laboratory, where they were dissected, and the fiber cells were separated from ovules by forceps; developing fibers were immediately frozen with liquid nitrogen and stored at −80 °C until RNA extraction. The concentration of each RNA sample was determined using a NanoDrop 2000 spectrophotometer (NanoDrop Technologies Inc., Wilmington, DE). RNA isolation and sequencing of samples were conducted as previously described (Naoumkina et al., 2024). Briefly, the Sigma Spectrum Plant Total RNA Kit (Sigma-Aldrich, St. Louis, MO, USA) was used for total RNA isolation. The concentration of each RNA sample was determined using a NanoDrop 2000 spectrophotometer (NanoDrop Technologies Inc., Wilmington, DE). The paired-end Illumina platform (PE150) was used for sequencing samples. Novogene Corporation (Chula Vista, CA, USA) performed library preparation and sequencing. Over 20 million paired raw reads were obtained for each RIL of the MAGIC population [34].

### DNA sequence analysis

Sequence reads were aligned to the JGI v 3.1 *G. hirsutum* reference genome [35] using hisat2 software and default parameters [36]. DNA variation was called by using bcftools mpileup software to read the bam files and generate the variant call format (vcf) files [37]. We filtered for high-quality SNP variants with a threshold of QUAL > 500. SnpEff software was used to annotate the vcf files with predictions of HIGH|MODERATE|MODIFY|LOW effects including nonsynonymous changes to the coding sequences, and changes to splice junctions and untranslated regions [38]. We then used grep -E ‘HIGH|MODERATE ‘software to filter the vcf files for lines that had been annotated by SnpEff as HIGH or MODERATE predicted effect on gene function. Further, we filtered with grep -E ‘1/1:’ to choose lines of the vcf file in which at least one of the newly sequenced lines had been scored as homozygous for the alternative allele (1/1: ALT). The HIGH|MODERATE variants were collected along with all variants near the genes that are highlighted in the main text to produce a vcf file of the genotypes of the 132 lines of historic cotton germplasm at the selected loci. [Supplemental Table S5].

### RNA sequence analysis

Sequence reads were aligned to the JGI v 3.1 *G. hirsutum* reference gene coding sequences (CDS) [35] using hisat2 software and default splice-aware parameters without further filtering [36]. Reads that mapped to CDS were counted using the idxstat module in samtools software [37] and normalized by the reads per kilobase per million reads (RPKM) approach [39]. For both developmental timepoints (8, 16-DPA) and for each gene, the expression of the 550 RILs was analyzed to identify bimodal distributions indicating potential expression QTLs (eQTLs) by a simplified approach. We computed the average RPKM of the lowest decile of RILs and the top decile. We computed the percent standard deviation and ranked the genes. For the purpose of transcriptome mining, we chose to focus on the ~ 5% of genes with the highest transcript expression variability. We noticed empirically, in this population, that the ~ 5% of genes with greater than 45% standard deviation in the distribution of RNA expression in the 550 MAGIC RILs were likely to appear bimodal, and we used this threshold for filtering our lists of candidate genes. We also required that the top decile of each gene be expressed > 3 RPKM in developing fiber tissue.

### Diversity of alleles at the qA07-STR locus

**Fig. 1:**
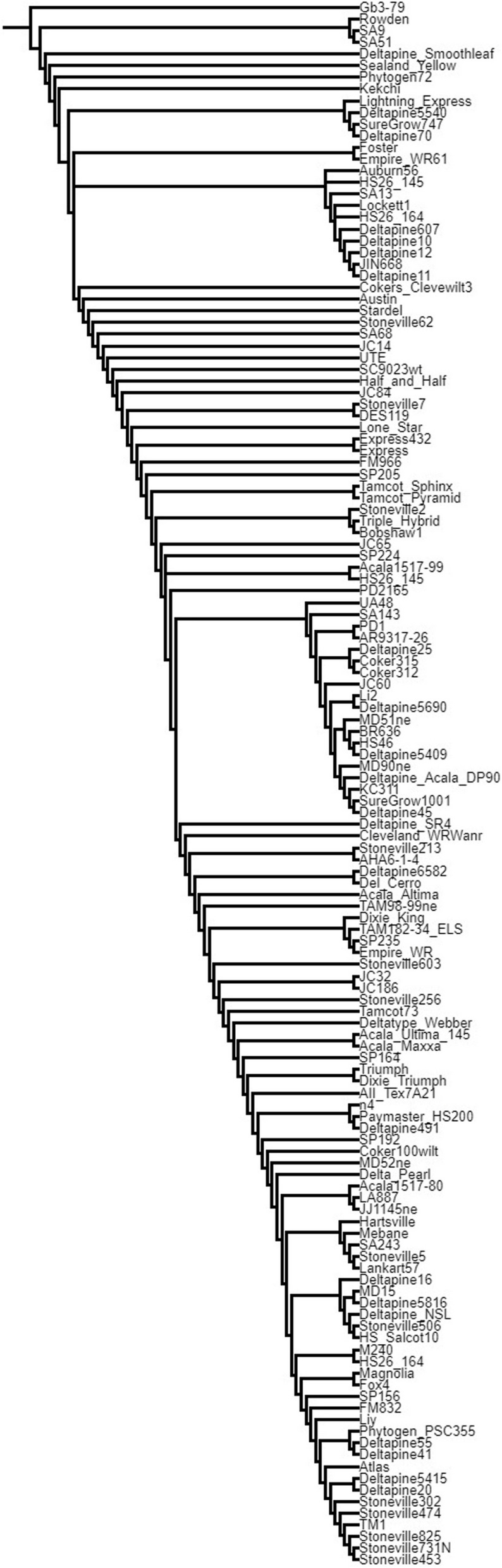
Diversity at qA07-STR cotton locus. The phylogenetic tree of the newly sequenced 132 *Gossypium hirsutum* lines based on the A07:60–99 Mb chromosome arm that contains a major cotton fiber strength (STR) QTL. See also Supplemental Table S1 for the Newick (nwk) formatted file

**Fig. 2:**
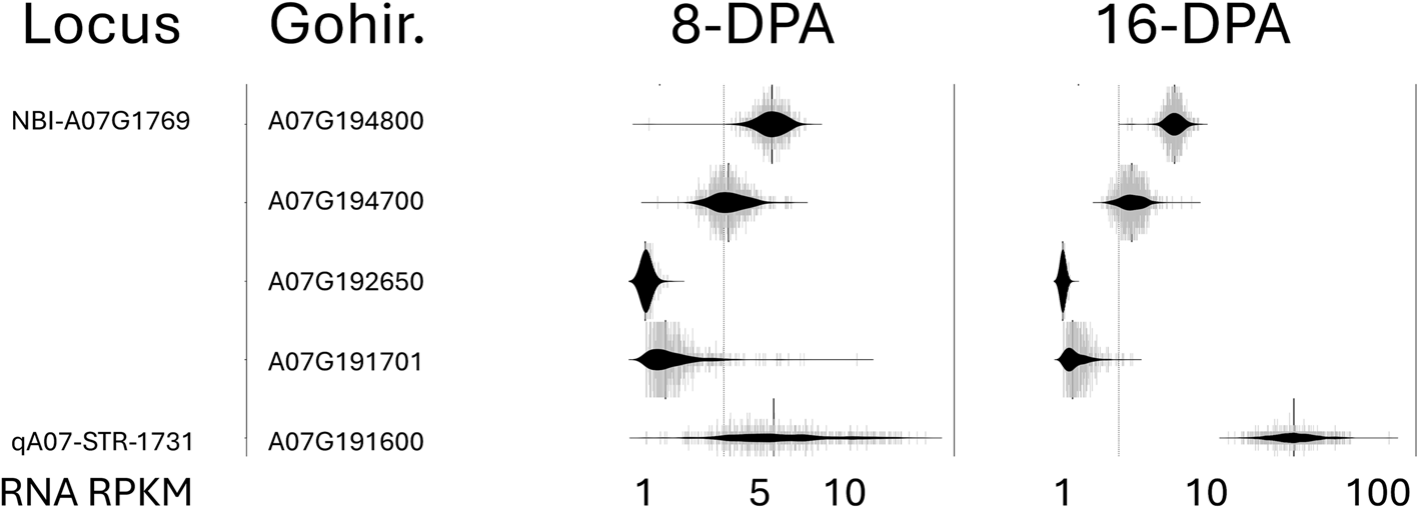
Expression of genes with non-synonymous variants near the qA07-STR locus. RNA expression (log(RPKM + 1)) of select genes at the qA07-STR locus for 550 MAGIC RILs at two developmental timepoints, 8 days-post-anthesis (DPA) and 16-DPA. See also Supplemental Tables S14 and S15 for all plotted data

We considered the qA07-STR locus that has been reported by our group and many others as highly significant as a positive control for our multi-mining method [33, 40–44]. We reasoned that if the filters we applied to DNA and RNA variation in our data could find a candidate gene at this locus, the other genes that passed the same filters would be of interest to cotton fiber breeders and researchers. To understand the history and diversity of genes at the qA07-STR locus that we identified as a major fiber strength QTL [32, 33, 45] in the germplasm we constructed a phylogenetic tree based on the chromosome arm at A07:60–99 Mb. We filtered for variants in genes of this region in the vcf file of the 132 cotton lines. Using TASSEL software, [46] a distance matrix was computed and a UPGMA Newick tree was generated. [Fig. 1, Supplemental Table S1] We rooted the tree with *G. barbadense* GB 3–79 reference sequence [47]. We further investigated the five genes predicted by SnpEff software to contain non-synonymous variation for diversity of RNA expression in the 550 MAGIC lines on the narrower A07:88–92 Mb interval based on recent fine mapping [Fig. 2] [48].

### Alignment of potential transposable element TE0076021 at the qA07-STR locus

We aligned the sequence of the 277 bp annotated putative transposable element (TE) TE0076021 [Supplemental Table S6] to the JGIv3.1 *Gossypium hirsutum* reference genome, the JGI *Gossypium raimondii* genome (D5), the WHU *G. hirsutum* and *G. arboreum* (A2) reference genomes using BLAST (-task blastn-short) at CottonMD to identify potential additional copies of the sequence [3]. Further we used BLAST at NCBI to search for potentially related orthologous sequences in other organisms [49].

### Potential flammability genes

We searched for variations in genes that are family members of the 13 genes that were suggested to influence the combustion of samples of fibers and the resulting textiles [27]. We used the previously suggested 13 genes to do a BLAST [49] search for homeologs and gene family members that are 90% identical to the query. These 278 genes and their annotations are listed in [Supplemental Table S7]. This list was compared to the lists of genes identified by the DNA and RNA variation.

### Plant phenotyping

The phenotypic data for the MAGIC RILs were previously reported [33]. Briefly, the eleven parents and subsets of the RILs were planted in Florence, SC, in 2014–2016, Starkville, MS, in 2009–2011, and 2014–2016, and in Stoneville, MS, in 2013–2015. Single row plots were 12 m long with approximately 120 plants per plot and two replicate plots per line at each location–year. Field practices were applied according to the prevailing conventions at each location–year. Twenty-five naturally opened bolls were manually harvested from each line and ginned using a 10-saw laboratory gin. The fiber quality attributes (ELO, MIC, SFI, STR, UHML, and UI) were measured using an HVI (USTER Technologies, Charlotte, NC, USA). Raw phenotypic data were then also normalized across replicates, years, and locations using a best linear unbiased predictor (BLUP) implemented in R software using the lme4 package to fit the model: “model = lmer(phenotype ~ (1|line) + (1|location) + (1|year) + (1|(replicate: location):year) + (1|line: location) + (1|line:year))”. [33, 50]

## Results

### Sequencing of 132 Gossypium lines

To identify novel DNA sequence variations that exist in the historic breeding lines, we obtained 20 × coverage of short read sequence data and aligned to the JGI v3.1 reference genome [35]. We identified 24,996 DNA sequence variations [Supplemental Table S5] that were predicted by SnpEff software to affect the function of 10,418 annotated genes that are expressed at either 8-DPA or 16-DPA [Supplemental Table S8].

### Transcriptome analysis

To identify gene expression variants that are segregating in the MAGIC population, we sequenced RNAs isolated from developing fibers of 550 RILs at 8-DPA and 16-DPA. The reads were aligned to the JGI v3.1 reference genome and expression was evaluated for genes with bimodal expression. We identified 2,517 fiber expressed genes at 8-DPA and 2,729 at 16-DPA, with suggestions of bimodal distributions in the population at the chosen > 45% standard deviation capturing the top ~ 5% of variable genes with fiber expression. [Supplemental Tables S9, S10] We did not observe any significant effect of planting year on the range of variability for the qA07-STR or qD13-STR candidate genes by ANOVA, and combined the data from RILs that had been planted over multiple years [Supplemental Tables S3, S4].

### Variation at the qA07-STR region

We were especially focused on a region where a major cotton fiber strength locus has been identified [32, 33, 40, 41, 45, 51, 52]. We used PCR-based markers to identify 12 haplotypes [Supplemental Table S11] in 43 of the lines [Supplemental Tables S12, S13]. The eleven parents that were used to construct the MAGIC RILs population contain 4 of these haplotypes, with Acala Ultima likely heterogenous in the ten pooled seedlings. We constructed a phylogenetic tree based on the whole genome DNA sequencing variants on the ChrA07:60–99 Mb region to observe how the haplotypes are dispersed through the historic breeding lines [Fig. 1]. The Newick tree formatted text string is included in [Supplemental Table S1] and can be inspected in a Newick tree viewer to observe branch lengths (eg. https://icytree.org) [53]. We identified five genes with non-synonymous DNA variants in the germplasm on this interval that are predicted to affect gene function [Fig. 2]. We explored the RNA expression of these five genes in the developing fiber transcriptomes from 8-DPA and 16-DPA of the MAGIC RILs [Fig. 2]. Notably, one of the genes has a bimodal, violin shaped distribution of gene expression, “A07-1731” (Gohir.A07G191600; Gh_A07G1731), suggesting differences in promoter activity or RNA transcript stability in the MAGIC RILs**.** The data plotted in Fig. 2 are presented in [Supplemental Tables S14, S15].

### Transposon promoter indel in A07-1731

Further scrutiny of the A07-1731 (Gohir.A07G191600) promoter sequence revealed a 277 bp indel, which is an insertion relative to the JGI v 3.1 reference. We assembled the insertion from the mapped reads and their unmapped read pairs and searched for a reference sequence that contains the insertion. The WHU *G. hirsutum* reference genome [3] has the correct insertion relative to the JGI reference and has annotated it as a transposable element TE0072061. [Figure S1]. The 277 bp sequence is 82% AT nucleotides [Supplemental Table S6]. We found that in both the JGI and WHU reference genome, a highly homologous sequence is present in the promoter of the D07 homeolog of the gene A07-1731, namely Gohir.D07G197900. The extant progenitor diploid *Gossypiums* also contain a similar sequence in the promoters of the orthologous genes Gar07G25470 [A2-WHU] and Gorai.001G221500 [D5-JGI] [3]. We found 22 BLAST hits in the JGI genome that are longer than 100-bp and > 80% identical in the introns and untranslated regions of annotated genes [Supplemental Table S16], but no hits longer than 18-bp within coding sequences [Supplemental Table S17]. We found 53 hits greater than 200-bp and > 80% identity in the full JGI *G. hirsutum* reference genome [Supplemental Table S18]. Further BLAST analysis revealed no clear orthologs in other species, or evidence to link this sequence to a known TE family [49].

### Nuclear export signal (NES) disrupted in A07-1731

We investigated the sequence variation in A07-1731 and identified that one of the alleles contains a SNP in the coding sequence that results in a non-synonymous substitution (C > T at A07:89,996,543). Since the L > F appears in the context of a LxxxLxxLxL motif, we considered that it might represent the disruption of a canonical nuclear export signal motif that is present in all eukaryotes and regulates signaling events [54–57]. We used NetNES 1.1 software to detect an NES in both alleles [58]. The reference type allele is predicted to contain an intact NES, while the alternative allele does not [Figure S2].

### Additional candidate genes from allele and transcriptome mining

**Fig. 3:**
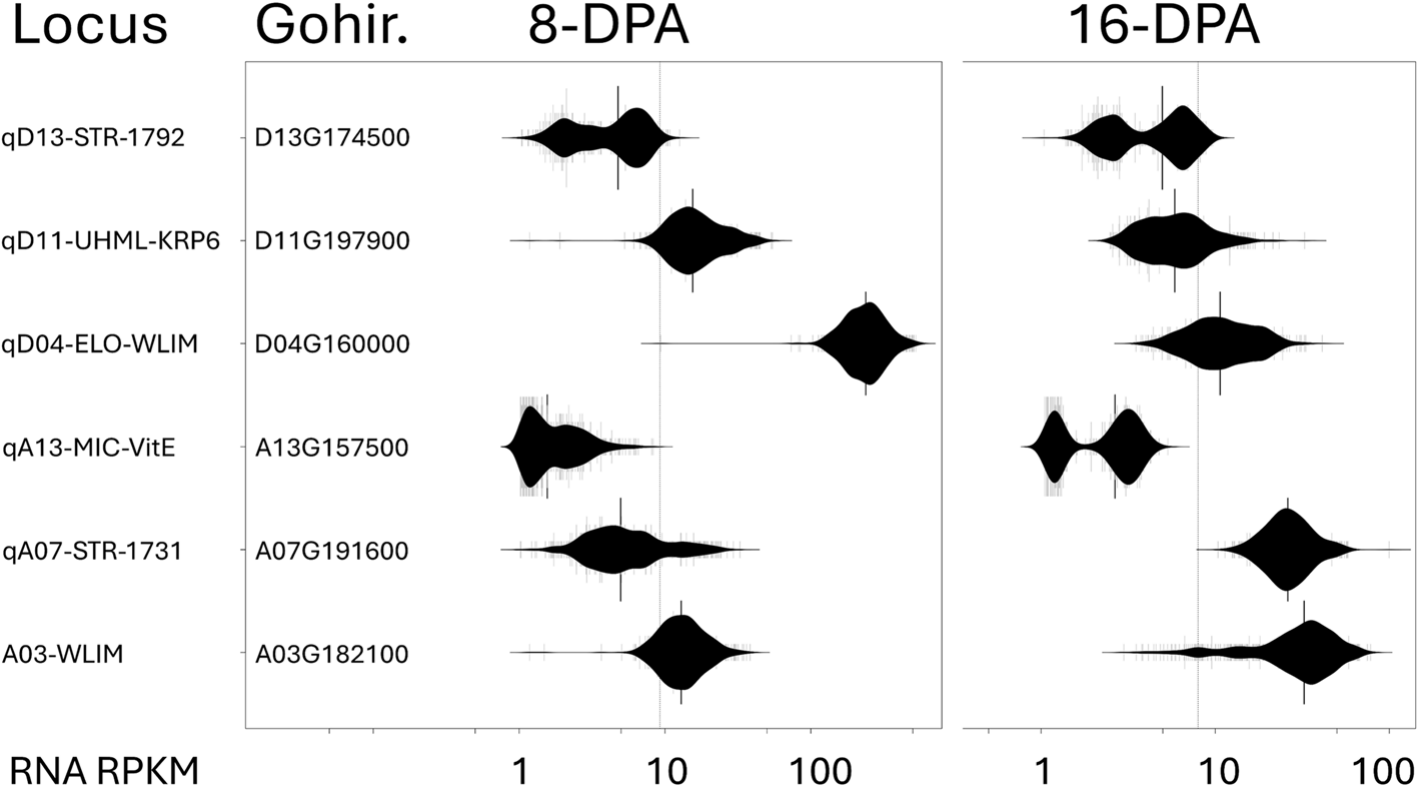
Fiber expressed genes with DNA and RNA variability near previously described QTL. RNA expression (log(RPKM + 1)) of select genes identified by the multi-mining of DNA variability in the historic germplasm and RNA expression variability in the 550 MAGIC RILs at 8- and 16-days post anthesis (DPA). See also Supplemental Tables S14 and S15 for all plotted data

Among genes that were predicted to have consequential DNA variation in the historic germplasm, we searched for genes that showed diverse RNA expression in the developing fibers of 550 MAGIC RILs. Notably, these criteria found two candidate genes that we have already reported, qD11-UHML-KRP6 (Gohir.D11G197900; Gh_D11G1929) and qD13-STR-1792 (“D13-1792”; Gohir.D13G174500; Gh_D13G1792). Another newly identified candidate for a fiber quality trait is very close to a qA13-MIC (“A13-VitE”; Gohir.A13G157500, Gh_A13G1336). Close to the qD04-ELO loci that we have previously reported [33] we identified a “D04-ELO-WLIM” (Gohir.D04G160000; Gh_D04G1562). We noticed another WLIM transcription family member, “A03-WLIM” (Gohir.A03G182100; Gh_A03G1598) with bimodal RNA expression and predicted consequential DNA variation. We also observed promoter indels at the A03-WLIM, A13-VitE and D13-STR-1792 loci. [Figs. 3, S3, S4] The data plotted in Fig. 3-HVI are presented in [Supplemental Tables S14 and S15].

### Potential flammability genes

**Fig. 4:**
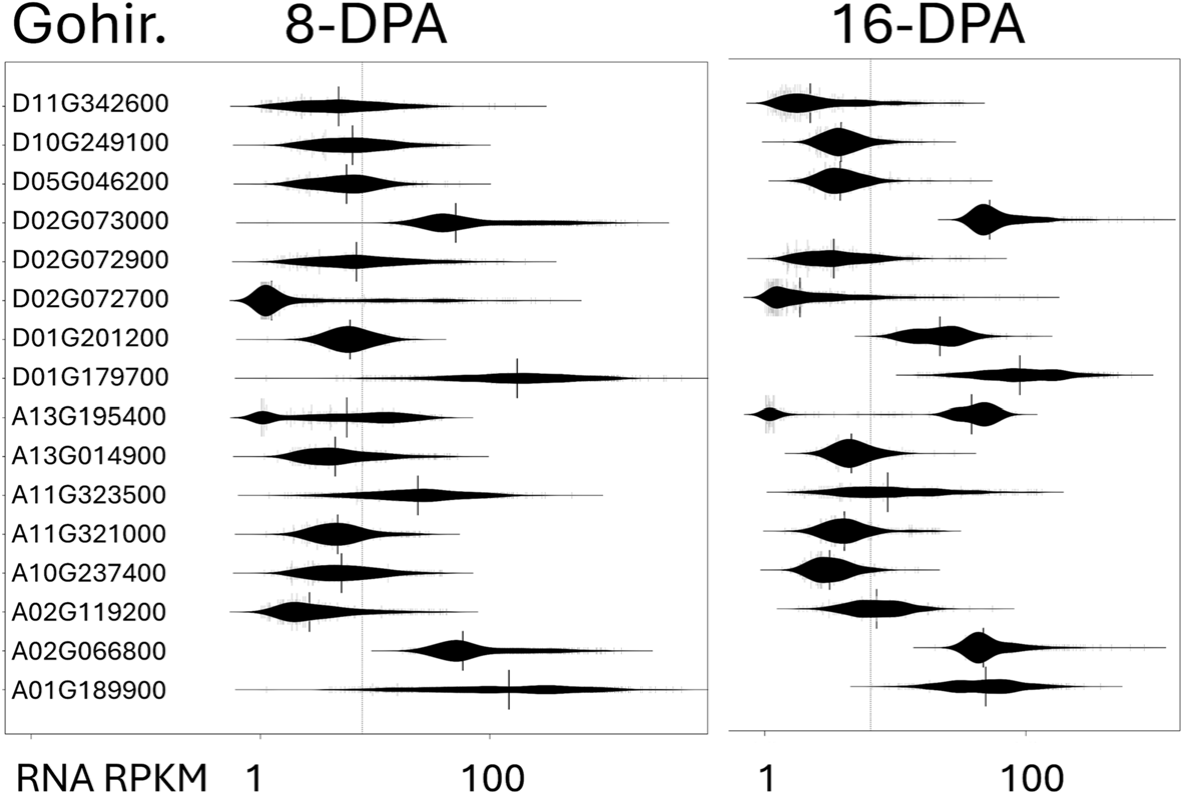
Potential flammability genes. RNA expression (log(RPKM + 1)) of select genes identified by the multi-mining of DNA variability in the historic germplasm and RNA expression variability in the 550 MAGIC RILs at 8- and 16-days post anthesis (DPA). These genes were also mined based on homology to potential cotton textile flammability genes. See also [Supplemental Table S7] for the larger list of potential flammability genes based on homology to the previously reported candidates. See also [Supplemental Tables S14 and S15] for all plotted data

We searched further for variations in genes that are family members of genes that were suggested to influence the combustion of samples of fibers and the resulting textiles [27]. We found 16 that have bimodal RNA expression in both 8 and 16-DPA fibers of the MAGIC RILs suggesting the possibility of useful expression variants [Fig. 4-FR, Supplemental Table S7].

### Epistatic interaction between qA07-STR with qD13-STR candidate genes

**Fig. 5:**
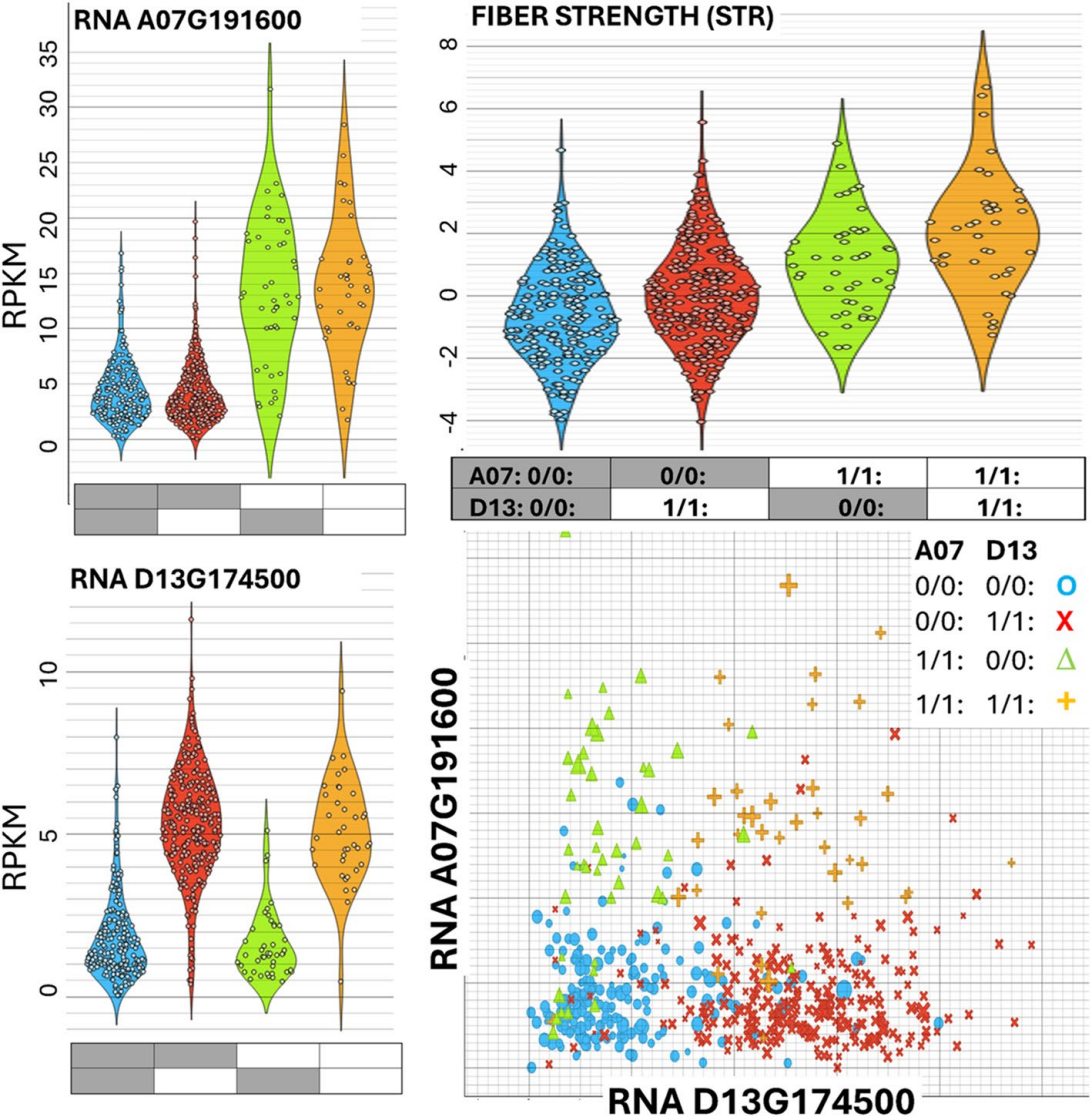
Additive epistasis between qA07-STR-1731 and qD13-STR-1792. Four combinations of alleles are presented with gray boxes indicating the JGIv3.1 reference allele (0/0:, REF) and white boxes indicating the alternative allele (1/1:, ALT). The ALT allele of the candidate genes qA07-STR-1731 and qD13-STR-1792 have higher RNA expression at 16-DPA than the REF alleles. qA07 and qD13 have an additive epistatic effect on cotton fiber strength (STR). Violin and scatter plots show 550 MAGIC RILs phenotypes and RPKM expression at 16-DPA for the four combinations of the alleles of the two candidate STR genes. See also [Supplemental Tables S14 and S19] for all plotted data, and [S20] for ANOVA statistics

To explore the consequences of the potential epistatic interaction between A07-1731 and another candidate fiber strength gene, D13-1792, on fiber phenotypes we divided the RILs based on their genotypes at both loci. The genotypes of each influence transcript abundance, with higher expression in RILs with the alternative allele of each. We found that there was an additive effect on fiber strength (STR) [Fig. 5]. The data plotted in Fig. 5 are presented in [Table S19] and were analyzed by ANOVA in [Supplemental Table S20]. Compared to the highest strength genotype A07-D13-1/1: 1/1:, the loss of only the ALT allele of D13 (ie. A07-D13-1/1: 0/0:) is significant at p < 0.01, and the loss of the ALT allele of A07 (ie A07-D13-0/0: 1/1:, or A07-D13-0/0: 0/0:) is significant at p < 0.001).

## Discussion

### Multi-mining of DNA and RNA variation

We were able to extend our research of cotton fiber trait genes by sequencing 132 historic cultivars and breeding materials. We identified novel variations in 10,418 genes that we are predicting to affect gene function of fiber expressed genes [Supplemental Tables S8]. We used transcriptomes from 8- and 16-DPA developing fibers from a population of 550 MAGIC RILs that descends from eleven of the lines in the historic germplasm [Supplemental Tables S9 and S10].

Previously, we had identified several fiber trait QTLs and candidate genes using the MAGIC RILs and a GWAS approach, and additional crosses for fine mapping [33, 41, 45].

With the addition of transcriptome data, we attempted to identify new candidate genes at those QTLs and to identify new genotypic diversity in fiber-expressed genes.

By taking together these new transcriptomes, new genomes and prior knowledge of QTL loci and already described gene families we performed allele mining and transcriptome mining.

### qA07-STR

The phylogeny based on the qA07 strength locus revealed new haplotypes that were not present in the eleven parents that founded the MAGIC population of 550 RILs [Supplemental Table S11]. These new haplotypes represent a source of diversity for this well-established strength locus. Further, the deeper sequencing and updated reference genome allowed us to identify variants that had been overlooked. Here, we report that the candidate gene A07-1731 has two alleles, the alternate one of which has an annotated transposable element insertion TE0072061 in the promoter and a disruptive nonsynonymous variant (C > T; L > F) in the putative nuclear export signal (NES) motif at A07:89,996,543 [Supplemental Figure S2]. The presence of the alternative allele is correlated with higher A07-1731 expression at both 8 and 16-DPA [Fig. 5, Supplemental Table S19]. This alternative strength allele is present in 24 of the 132 sequenced lines, as observed for the NES SNP at A07:89,996,543 [Supplemental Table S21].

The role of the NES in A07-1731 in signal transduction has not been shown for this gene experimentally and is a speculative mechanism for the STR fiber phenotype. Since the identification of the NES motif in the 1990’s many other genes with the motif that allows CRM1-mediated nuclear transport have been identified [59]. The canonical NES sequence follows a loose consensus sequence and is important for signal transduction across all eukaryotes including plants and humans[54–57, 60, 61]. More than 200 of these canonical NES have been experimentally validated and are available at the NESdb [62] [http://prodata.swmed.edu/LRNes]. Since this L > F substitution preserves the hydrophobic side chain at this residue it may not disrupt the ability of the A07-1731 to interact with CRM1 and exit the nucleus [63].

The role of promoter indels in transcriptional diversity has been well established in all organisms [64]. The annotation of the 277-bp indel in the promoter of the A07-1731 candidate strength gene as a transposable element lacks further support. The sequence is present in both of the extant diploid progenitors (D5 and A2) of the tetraploid *G. hirsutum* (AD1). Both the JGI and WHU reference genomes contain the homeologous sequence in the D07 chromosome, in the promoter of the D07 homeolog (Gohir.D07G197900, Ghi_D07G10961) of the A07-1731 gene. Only the WHU reference contains the sequence in the promoter of the candidate qA07-STR gene (Gohir.A07G191600, Ghi_A07G11716). The presence of the sequence is correlated with increased expression of the A07-1731 gene in the MAGIC RILs and increased cotton fiber strength [Fig. 5-ST, Supplemental Table S19]. Due to the high AT nucleotide content (82%) of this 277-bp sequence, we speculate that the low melting temperature of the motif allows greater access to the promoter by the transcriptional apparatus resulting in increased expression and subsequently increased fiber strength. The comparison of reference *Gossypium* genomes suggests that the sequence was present in the promoter of the progenitor A and D genomes and was lost in some lines of the tetraploid *G. hirsutum*, perhaps due to DNA polymerase slippage on the AT-rich sequence [65]. Since 24 of the 132 lines sequenced contain the same 277-bp deletion and NES variant, we expect that this haplotype originated before or early in the breeding programs represented in our germplasm collection.

On the A07:88–92 Mb region, we presented the RNA abundance in candidate genes that have been previously suggested. Several previously suggested qA07-STR candidate genes including Gohir.A07G194800 (NBI-Gh_A07G1769) that we predict to have consequential DNA variation do not show significant variance in the RNA expression in the developing fibers of the segregating MAGIC RILs [Fig. 2] [51, 52]. Similarly, Gohir.A07G192650 (NBI-Gh_A07G1744) has been suggested before but appears to have normal distributions of RNA expression in the MAGIC RILs [Fig. 2] [42, 66, 67]. Of these five candidates near qA07-STR, only A07-1731 (Gohir.A07G191600; Gh_A07G1731) has a bimodal distribution of RNA expression in developing fibers in the segregating MAGIC RILs. Recent marker analysis of this locus on advanced populations has narrowed the candidate interval and still includes A07-1731 [32, 45, 48].

### Additive epistasis between qD13-STR-1792 and qA07-STR-1731 for fiber strength

Among the genes that our multi-mining approach identified is “D13-1792” (Gohir.D13G174500; Gh_D13G1792). We previously identified an allele of this gene that contains a premature stop codon [33]. We and other groups have provided evidence of association with cotton fiber strength [33, 52, 66]. Using the GWAS of MAGIC RILs, we unmasked qD13-STR-1792 after excluding the 10% of MAGIC RILs that had the desirable alternate allele at the qA07-STR-1731 locus [33]. We here present transcript and phenotype evidence to support the additive epistatic interaction between these two genes on the cotton fiber strength phenotype. The expression of both genes is controlled independently by the allele variant, with (1/1: ALT) alleles showing higher RNA expression, without a significant effect of the combination of A07-1731 and D13-1792 alleles on each other [Fig. 5, Supplemental Tables S19 and S20]. However, the HVI phenotypes show that the combination of genes has an additive effect on cotton fiber strength (STR). D13-1792 is annotated as an ankyrin repeat (ANK) family protein. The characteristic ~ 33 residue ANK motif is present in hundreds of proteins across the diversity of life, from viruses to humans, and is considered to be a mediator of protein–protein interactions [68]. The diverse interactions of ANK proteins have implicated them in cytoskeleton integrity, plant immunity, transcriptional regulation, and signal transduction [69]. The orthologs of A07-1731 are not well described. The ortholog in Arabidopsis is predicted to be a transmembrane protein, located in the cytosol that can form a homodimer (AT2G04515; https://www.arabidopsis.org/locus?key=500230869). Physical interactions between D13-1792 and A07-1731 may orchestrate signals including nuclear-cytoplasmic shuttling that may contribute to the cotton fiber strength phenotype but have not yet been investigated.

### qA13-MIC

Here we identify a candidate gene, “A13-VitE” (Gohir.A13G157500; Gh_A13G1366) with a variable promoter sequence [Supplemental Figure S4] close to the previously reported qA13-MIC locus [33, 70]. The promoter indel clearly affects the expression of the gene. [Fig. 3]. This tocopherol O-methyltransferase/gamma-tocopherol methyltransferase likely affects the accumulation of vitamin E, an important antioxidant. A key role of free radicals and mitochondrial metabolic function has been implicated in the development of cotton fiber maturity [71–73]. Interestingly, vitamin E has been studied as protective against gossypol toxicity, which is a toxin present in cotton seed that is harmful to animals and humans if ingested [74]. Further work is warranted to characterize the molecular function of this candidate gene and its role in cotton fiber fineness and cotton seed nutritional feed value.

### qD04-ELO-WLIM and A03-WLIM transcription factors

We identified two WLIM transcription factor genes with both DNA and RNA variability, “D04-WLIM” (Gohir.D04G160000; Gh_D04G1562) and “A03-WLIM” (Gohir.A03G182100; Gh_A03G1598). Previously, another cotton WLIM1a was demonstrated to have dual functions in fiber cell elongation and secondary cell wall formation [8]. WLIM1a (JX648310, Gohir.D06G021500) was shown by both gain and loss of function experiments to alter the architecture of the secondary cell wall, resulting in longer and thicker fibers. The thickness and secondary cell wall structure of fiber cells are directly related to fiber strength and fineness [4, 75]. The activity of a WLIM1a as a transcription factor was previously shown in addition to an actin bundling function suggested by co-precipitation of WLIM1a with F-actin [8].

D04-WLIM has a sequence variability in the 3’UTR in the diversity panel and MAGIC RILs that is predicted to affect the stability of the transcript [Supplemental Table S5]. This gene is located at a major qD04-ELO QTL [33].

A03-WLIM was predicted by SnpEff to have variation in the sequenced lines that affect its promoter, including a 32-bp indel at JGI-A03:108,161,215. [Supplemental Figure S3].

### qD11-UHML

The fiber length candidate gene at qD11-UHML-KRP6 (Gohir.D11G197900; Gh_D11G1929) has been identified and characterized as a KRP6 gene by our group and others [31, 33, 41, 76]. Further work to establish a relationship with an E3-ligase signal transduction gene on Chr.D12 was recently reported [34]. Here, we find the gene again by a new approach, transcriptome mining for segregating transcript expression in an informative population. However, rather than finding it based on phenotype, we found it by the bimodal, violin shaped plots of the expression in the 550 MAGIC RILs developing fibers. [Fig. 3].

### Flame retardancy and cotton genetics

The flammability of textiles is influenced by the genetics of the cotton plant. Recently, several of the MAGIC RILs were observed to produce self-extinguishing textiles [27]. Since the parents of the MAGIC population are present in the diversity panel, the previously identified candidates could be identified again. Further, we BLASTed the candidate genes to identify gene family members. This larger list of 278 genes [Supplemental Table S7] was compared to the lists of genes from allele and transcriptome mining. Of these, we found 16 genes with RNA variation in the MAGIC RILs at both 8 and 16-DPA [Fig. 4]. Although we have not yet screened or evaluated these historic germplasm, we are optimistic that the success of the multi-mining approach to find gene variants that have already been characterized, suggests that some of the sixteen genes that we highlighted and the variant call vcf file [Supplemental Table S5] we provide can direct breeders and researchers more directly to new knowledge than doing an exhaustive screen without the benefit of prior knowledge including DNA and RNA variability.

## Conclusion

A transcriptome treated as a quantitative phenotype is used to identify eQTLs. Here we took a simplified approach to identify genes with bimodal expressions in the MAGIC population. The genes that we highlighted in this paper meet the four criteria of our multi-mining approach: DNA variability; RNA expression in fiber tissue; variability of RNA expression in a segregating population; and precedent in the literature for a gene family’s involvement in the developmental process of the cotton fiber cells. The newly sequenced historic cotton germplasm revealed the diversity of novel haplotypes and candidate genes at several know QTL. We expect that in the attached vcf file, other researchers will find useful variants in the 10,418 cotton fiber variant genes we mined [Supplemental Table S5].

## Supplementary Information

Supplementary Material 1: Supplemental Figure S1. Synteny and diversity among NBI, HAU, WHU and JGI reference genomes at “A07-1731” (Gohir.A07G191600) locus reveals a promoter indel annotated as transposable element TE0072061 in the WHU genome, that we also observed segregating in our historic germplasm and MAGIC RILs in association with cotton fiber strength (STR)

Supplementary Material 2: Supplemental Figure S2. Disruption of canonical LxxxLxxLxL nuclear export signal motif (NES) due to a L>F amino acid substitution in the desirable, high fiber strength (STR) cotton lines and MAGIC RILs is predicted by NetNES 1.1 software

Supplementary Material 3: Supplemental Figure S3. Synteny and diversity among NBI, HAU, WHU and JGI reference genomes reveals a promoter indel near the qA13-MIC QTL, that we also observed segregating in our historic germplasm and MAGIC RILs

Supplementary Material 4: Supplemental Figure S4. Synteny and diversity among NBI, HAU, WHU and JGI reference genomes reveals a promoter indel at the candidate gene for the qD13-STR-1792 QTL, that we also observed segregating in our historic germplasm and MAGIC RILs

Supplementary Material 5: Supplemental Figure S5. Synteny and diversity among NBI, HAU, WHU and JGI reference genomes reveals a promoter indel at A03-WLIM that we also observed segregating in our historic germplasm and MAGIC RILs

Supplementary Material 6: Supplemental Table S1. Newick tree and taxa list for Gossypium hirsutum historic germplasm and select breeding lines from the USDA germplasm that were sequenced for this present manuscript. NWK formatted string text can be further inspected with a Newick tree viewer (eg. https://icytree.org). Supplemental Table S2. Planting years for the MAGIC RILs that were grown for isolation of RNA from 8- and 16-DPA fiber cells. All plants were grown in the same field in New Orleans, LA, USA. Supplemental Table S3. ANOVA statistics that show that the year-to-year effect of RNA expression of the A07-1731 (Gohir.A07G191600) gene is negligible. Supplemental Table S4. ANOVA statistics that show that the year-to-year effect of RNA expression of the D13-1792 (Gohir.D13G17450) gene is negligible. Supplemental Table S5. Variant Call Format (vcf) file annotated by SnpEff software for selected variants observed in the present germplasm. Supplemental Table S6. Fasta sequence of the 277-bp insertion that is annotated as transposable element TE0077021 in the WHU G. hirsutum reference genome and is associated with increased fiber expression of A07-1731 and increased fiber strength. Supplemental Table S7. Thirteen genes that were identified as potentially involved in textile flammability (Table 1, Table S10 in [27]) were BLASTed against the JGIv3.1 cotton reference genome to find homeologs and gene family members that have >90% identity to the prior candidates. The homologs of these 278 genes in three other cotton reference genomes are shown, along with the JGI annotations. Supplemental Table S8. 10,418 genes that are presented in the Supplemental Table S5 vcf file. The annotation columns indicate the potential flammability genes; the standard deviation (>45%) of fiber RNA expression in 550 MAGIC RILs at 8- and 6-DPA; and if the SnpEff software predicts consequential DNA variation in the historic germplasm. Supplemental Table S9. 2,517 genes that show more than 45% standard deviation in the 550 MAGIC RILs developing fiber RNA at 8-days post anthesis (DPA). Annotation columns show gene length; percent sandard deviation (pcSD); the average RPKM expression of the top decile (p90) and lowest decile (p10); SnpEff software prediction (see also Supplemental Table S5); and presence in the FR-300 potential flammability genes. Supplemental Table S10. 2,729 genes that show more than 45% standard deviation in the 550 MAGIC RILs developing fiber RNA at 16-days post anthesis (DPA). Annotation columns show gene length; percent sandard deviation (pcSD); the average RPKM expression of the top decile (p90) and lowest decile (p10); SnpEff software prediction (see also Supplemental Table S5); and presence in the FR-300 potential flammability genes. Supplemental Table S11. PCR-based SNP markers are shown for select, representative haplotypes from the sequenced lines including the parental lines of the MAGIC RILs to identify haplotypes at the qA07-STR-60-99Mb cotton fiber strength locus. Supplemental Table S12. PCR-based SNP markers were run on 43 of the sequenced G. hirsutum lines to identify haplotypes at the qA07-STR-60-99Mb cotton fiber strength locus. Supplemental Table S13. SNAPER-type PCR based SNP assay primers and an SSR-type length polymorphism primers. Supplemental Table S14. The data of select genes plotted in Figures 2, 3, 4 for RNA expression of 8-DPA fiber cells. P10 is the average expression of the bottom decile (10%) of RPKM values, P90 is the average of the top decile, PCSD is the percent standard deviation. Supplemental Table S15. The data of select genes plotted in Figures 2, 3, 4 for RNA expression of 16-DPA fiber cells. P10 is the average expression of the bottom decile (10%) of RPKM values, P90 is the average of the top decile, PCSD is the percent standard deviation. Supplemental Table S16. The BLAST results (-task blastn-short) of the putative transposable element TE0076021 that is in the promoter of A07-1731 (Gohir.A07G191600), in 24 of the 132 sequenced cotton lines, against the gene-space of the JGI reference genome, including coding sequences (CDS) and introns and untranslated regions (UTRs). Supplemental Table S17. The BLAST results (-task blastn-short) of the putative transposable element TE0076021 that is in the promoter of A07-1731 (Gohir.A07G191600), in 24 of the 132 sequenced cotton lines, against the coding sequences (CDS) of the JGI reference genome. Supplemental Table S18. The BLAST results (-task blastn-short) of the putative transposable element TE0076021 that is in the promoter of A07-1731 (Gohir.A07G191600), in 24 of the 132 sequenced cotton lines, against the whole genome of the JGI reference sequence. Supplemental Table S19. The data plotted in Figure 5. RNA expression of 16-DPA fiber cells for the A07-1731 and D13-1792 genes, fiber strength (STR) as measured by HVI, and genotypes of each RIL at both loci. Supplemental Table S20. Additive epistasis ANOVA test of the influence of the A07-D13 genotypes on fiber strength (STR-125). Supplemental Table S21. Select SNPs from Supplemental Table S5-VCF including the putative nuclear export signal (NES) variant at A07:89,996,543 in the A07-1731 strength (STR) gene. Of the 132 lines of historic and breeding germplasm, 24 are homozygous for the alternative (ALT, 1/1:) allele and 30 are heterogenous

## Data Availability

The filtered and annotated vcf and RNA variance tables are provided as supplemental tables [Tables S2, S6, S7]. These and all useful datasets generated for the scope of this study are included in the article or Supplementary Materials.
